# Election of Members

**Published:** 1867-07

**Authors:** 


ELECTION OF MEMBERS.

The following were also elected permanent members, by unanimous vote:—

Drs. John M. West, Williamsville.

Thos. Hickman, Vandalia, Fayette Co.

B. H. Cheeny, Joliet, Will Co.

Chas. Kerr, Pawnee, Sangamon Co.

J. Sweeney, Normal, McLean Co.

A. L. Kimber, Waverly, Morgan Co.

J. P. Mathews, Carlinville, Macoupin Co.

C. L. Hart, Irving, Montgomery Co.

John Walker, Berlin, Sangamon Co.

Henry W. Boyd, Alton, Madison Co.
J. L. Millier, Springfield, Sangamon Co.
J. C. Ross, Lincoln, Logan Co.
John W. Lawrence, Carbondale, Jackson Co.
G. H. Peebles, Williamsville.

